# Granzyme K mediates IL-23-dependent inflammation and keratinocyte proliferation in psoriasis

**DOI:** 10.3389/fimmu.2024.1398120

**Published:** 2024-06-05

**Authors:** Katlyn C. Richardson, Alexandre Aubert, Christopher T. Turner, Layla Nabai, Sho Hiroyasu, Megan A. Pawluk, Rachel A. Cederberg, Hongyan Zhao, Karen Jung, Angela Burleigh, Richard I. Crawford, David J. Granville

**Affiliations:** ^1^ International Collaboration on Repair Discoveries (ICORD) Centre, Vancouver Coastal Health Research Institute, University of British Columbia, Vancouver, BC, Canada; ^2^ Department of Pathology and Laboratory Medicine, University of British Columbia, Vancouver, BC, Canada; ^3^ British Columbia Professional Firefighters’ Burn and Wound Healing Group, Vancouver Coastal Health Research Institute, University of British Columbia, Vancouver, BC, Canada; ^4^ Integrative Oncology Department, British Columbia (BC) Cancer Research Centre, Vancouver, BC, Canada; ^5^ Department of Dermatology and Skin Sciences, University of British Columbia, Vancouver, BC, Canada

**Keywords:** granzymes, granzyme K, psoriasis, IL-23, PAR-1, inflammation, proliferation, skin

## Abstract

Psoriasis is an inflammatory disease with systemic manifestations that most commonly presents as itchy, erythematous, scaly plaques on extensor surfaces. Activation of the IL-23/IL-17 pro-inflammatory signaling pathway is a hallmark of psoriasis and its inhibition is key to clinical management. Granzyme K (GzmK) is an immune cell-secreted serine protease elevated in inflammatory and proliferative skin conditions. In the present study, human psoriasis lesions exhibited elevated GzmK levels compared to non-lesional psoriasis and healthy control skin. In an established murine model of imiquimod (IMQ)-induced psoriasis, genetic loss of GzmK significantly reduced disease severity, as determined by delayed plaque formation, decreased erythema and desquamation, reduced epidermal thickness, and inflammatory infiltrate. Molecular characterization *in vitro* revealed that GzmK contributed to macrophage secretion of IL-23 as well as PAR-1-dependent keratinocyte proliferation. These findings demonstrate that GzmK enhances IL-23-driven inflammation as well as keratinocyte proliferation to exacerbate psoriasis severity.

## Graphical Abstract

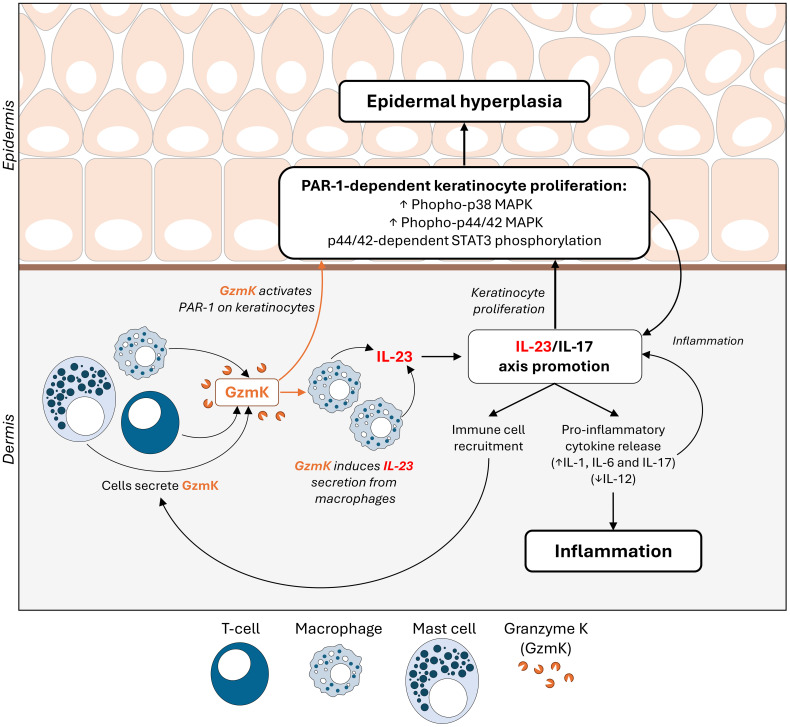

## 1 Introduction

Psoriasis is a chronic inflammatory disease that affects over 125 million people worldwide (1). Psoriasis vulgaris (plaque psoriasis) is the most common clinical variant, observed in 85-90% of psoriasis cases, and usually manifests as erythematous plaques covered with silvery scales on the extensor surfaces, trunk, and scalp (2). Persistent pruritus, pain, and visibility of plaques characteristic of the disease considerably impair quality of life (3, 4).

While biologics targeting the IL-23/IL-17 pathway have demonstrated clinical efficacy, their broad use is limited by costs, side effects related to immunomodulation, and disease recurrence following treatment discontinuation (5, 6). As such, an impetus to develop more affordable and efficacious therapeutics for psoriasis remains (7, 8). To address this need, a clearer understanding of the molecular mechanisms underlying pathogenesis is required.

Psoriasis pathogenesis is driven by elevated inflammation and keratinocyte proliferation. These processes are dependent on IL-23 and JAK/STAT signaling (9–12). During active disease, IL-23, produced by myeloid cells, activates and maintains the pathogenic phenotypes of Th17, Tc17, and possibly Treg cells, leading to the release of various pro-inflammatory cytokines, most notably IL-17 (13–15). This cytokine cascade fuels inflammation, perpetuating vasodilation and immune cell infiltration (9). Concurrently, STAT3 hyperactivation in keratinocytes drives increased cellular proliferation and impaired differentiation, leading to epidermal hyperplasia which presents clinically as skin plaques (9–12). Provided the central roles of IL-23 and STAT3 in psoriasis pathogenesis, exploring novel mediators within this context is imperative to advance our understanding of disease mechanisms and yield new opportunities for therapeutic development.

The immune cell-secreted serine protease, Granzyme K (GzmK), conventionally known for its role in targeted cell death, has emerged as a key player in inflammation (16). Elevated GzmK levels are consistently observed in biospecimens from individuals with diverse inflammatory conditions compared with healthy controls, including acute lung inflammation (bronchoalveolar lavage fluid) (17), endotoxemia (blood) (18), sepsis (blood) (19), viral infection (blood) (20), thermal injury (skin) (21), atopic dermatitis (skin) (22), and inflammaging (various tissues) (23). Corresponding to its presence in biofluids, extracellular roles for GzmK have been forwarded. Notably, GzmK has been shown to induce the release of pro-inflammatory cytokines (IL-1β, TNF-α, IL-6, IL-8, and monocyte chemoattractant protein-1 (MCP-1/CCL2)) (21, 24–26) and to promote cellular proliferation through activation of protease activated receptor-1 (PAR-1) (21, 24, 26, 27). Further, PAR-1-induced keratinocyte proliferation may contribute to psoriasis (28–31). Taken together, we hypothesized that GzmK contributes to inflammation and/or keratinocyte proliferation in psoriasis.

## 2 Results

### 2.1 GzmK is elevated in human psoriasis lesions and predominantly localized in the infiltrating immune cells of the papillary dermis

**Figure 1:**
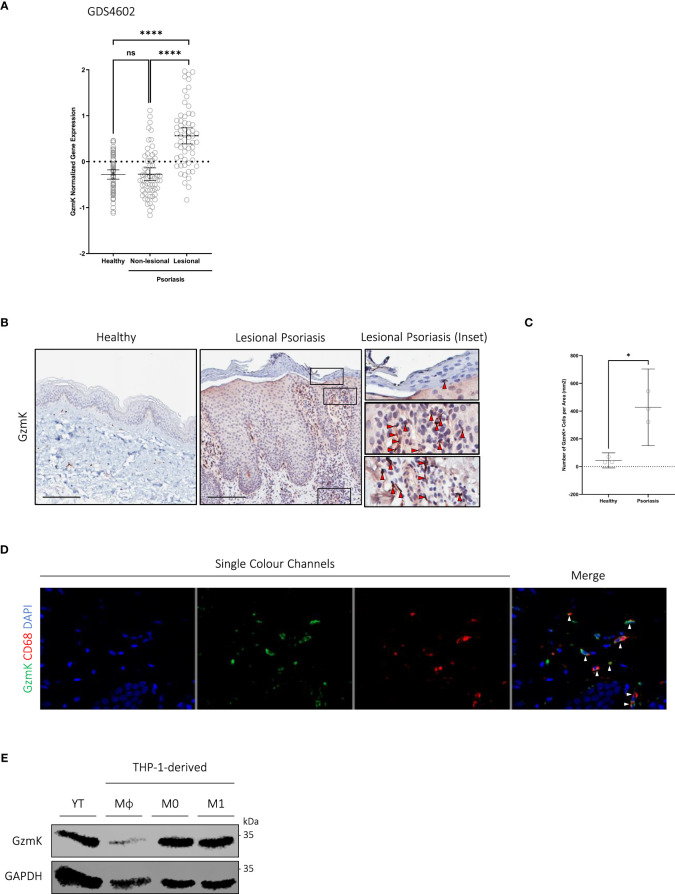
GzmK is abundant in human psoriasis lesions and predominantly localized in the infiltrating immune cells of the papillary dermis **(A)** GzmK normalized gene expression in the skin of healthy control subjects and psoriasis patients (both non-lesional and lesional). **(B)** GzmK immunostaining in the skin of healthy control subjects and psoriasis patients. The inset shows high magnification of regions of interest. Red arrows indicate GzmK^+^ cells. **(C)** Quantification of GzmK-positive cells presented as number of GzmK-positive cells per area (mm^2^) in the skin of healthy control subjects and psoriasis patients. **(D)** Two-colour immunofluorescence of GzmK and CD68 in psoriasis-affected human skin. ‘Merge’ showcases overlap of all colour channels. White arrows indicate co-staining cells (GzmK^+^/CD68^+^). **(E)** Western blot immunodetection of GzmK in NK cells (YT) and THP-1-derived monocytes (Mϕ), M0 and M1 macrophages. n ≥ 58 per group **(B–E)**. Data were analyzed by Kruskal-Wallis test with Dunn’s *post-hoc* test for multiple comparisons and presented as mean with 95% CI **(A)**, or Welch’s t-test and presented as mean with 95% CI **(C)**. In all plots, *P≤0.05, ****P≤0.0001. Scale bars represent 200 μm **(B)**, with representative images shown.

To evaluate GzmK levels in human psoriasis tissues, GzmK expression was assessed using microarray data available through the Gene Expression Omnibus (GEO, NCBI) database (32). According to dataset GDS4602 (33–35), GzmK mRNA expression is elevated in lesional psoriasis skin compared to both healthy control and non-lesional psoriasis skin (**Figure 1A**). Similar results were obtained using another dataset, GSD4600 (36, 37) (**Supplementary Figure 1A**). To further assess the presence of GzmK in psoriatic skin, a database query for GzmK was performed on skin mRNA microarray datasets publicly available on SEEK (Princeton University) (38). According to this search, the majority of prioritized datasets were related to psoriasis, underscoring the significance of GzmK to psoriasis pathology (**Supplementary Table 1**). Importantly, dataset enrichment analysis revealed ‘psoriasis’ as the disease category with the highest number of relevant datasets retrieved, further emphasizing its significance in the context of GzmK (**Supplementary Table 2**).

To examine GzmK at the protein level and its distribution in human psoriasis tissue, formalin-fixed paraffin-embedded sections of healthy control skin and lesional psoriasis skin were evaluated. In agreement with the GzmK mRNA expression data, all psoriasis skin samples exhibited a significant increase in GzmK protein levels compared with healthy control skin (**Figures 1B, C**). This increased GzmK detection occurred in all psoriasis samples despite reported differences in clinical appearance, sex, and age of individuals (patient data listed in **Supplementary Table 3**). Within psoriasis lesions, GzmK-positivity was also observed in the extracellular milieu surrounding the GzmK-positive cells compared with healthy controls (**Supplementary Figure 1B**), indicating potential extracellular secretion.

In all stained psoriasis lesions, GzmK-positive cells were primarily located within the inflammatory cell infiltrate of the papillary dermis and adjacent to small blood vessels (**Figure 1B**). In healthy skin, GzmK was detected almost exclusively in mast cells (Toluidine Blue O (TBO)^+^ and tryptase^+^), as previously reported (22). GzmK-positive mast cells were observed in psoriatic skin as well (**Supplementary Figures 1C–E**); however, there was a decrease in the percentage of GzmK-positive mast cells. Serial immunostaining and co-immunofluorescence of human psoriasis lesions further revealed a population of CD68^+^ cells (monocytes/macrophages) positive for GzmK (**Figure 1D**, **Supplementary Figure 2A**). We also detected a minor population of CD3^+^ cells (both CD4^+^ and CD8^+^ T cells) and Neutrophil Elastase (NE)^+^ cells (neutrophils) positive for GzmK (**Supplementary Figures 2A**, **3A**). No GzmK was detected in CD56^+^ (natural killer cells), CD1a^+^ (Langerhans cells), nor CD11c^+^ cells (dermal dendritic cells) despite each of these cell populations being elevated in human psoriasis lesions (**Supplementary Figures 2A**, **3A**). Further, human THP-1 monocytes and macrophages were confirmed as a source of GzmK *in vitro* (**Figure 1E**), corresponding with previous observations (21).

### 2.2 GzmK depletion attenuates disease severity in a murine model of IMQ-induced psoriasis-like skin inflammation

**Figure 2:**
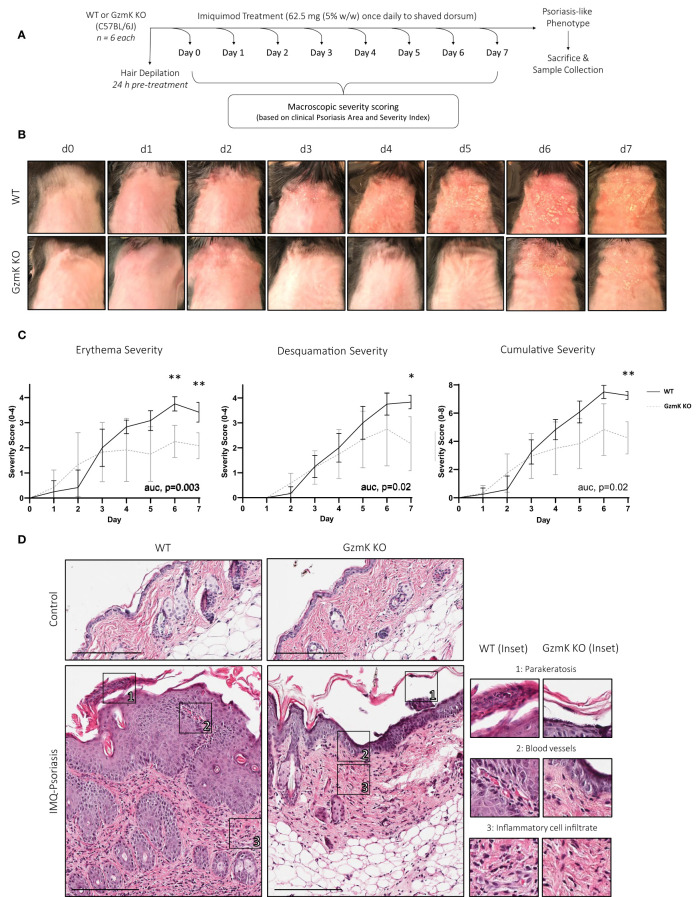
GzmK depletion attenuates disease severity in a murine model of IMQ-induced psoriasis-like skin inflammation. **(A)** Experimental protocol involving daily topical application of IMQ for 7 days to induce a psoriasis-like phenotype. **(B)** Images of IMQ-treated WT and GzmK KO mice from day 0 to day 7. **(C)** Daily changes in skin severity (erythema and desquamation) in IMQ-treated WT and GzmK KO mice. Cumulative skin severity is represented as cumulative erythema and scaling scores. **(D)** Hematoxylin and Eosin staining of paraffin sections from the dorsal skin of both IMQ-treated WT and GzmK KO mice. The inset shows high magnification of regions of interest. n ≥ 6 per group **(A-D)**. Data were analyzed by multiple unpaired t-tests with Welch correction and Holm-*Šídák post-hoc* test for multiple comparisons and presented as multivariable linear regression with three degrees of interaction **(C)**. In all plots, area under curve (AUC) p-values are as written and adjusted p-values are shown *P≤0.05, **P≤0.01. Scale bars represent 200 µm **(D)**, with representative images shown.

To investigate whether GzmK exerts a pathologic role in psoriasis, *GZMK* knockout (GzmK KO) mice were utilized. In comparative sequence analysis, human and mouse GzmK exhibited a 71.82% amino-acid sequence identity and full conservation of the catalytic triad (His, Asp, Ser) (**Supplementary Figure 4A**), underscoring the relevance of the mouse model for investigating the mechanistic role of GzmK in the context of psoriasis. Using the established murine model of imiquimod (IMQ)-induced psoriasis-like skin inflammation (39), a psoriasis-like phenotype was induced in both wild-type (WT) and GzmK KO mice (**Figure 2A**). WT and GzmK KO mice showed no visible signs of distress, and their body weights remained stable throughout the study (**Supplementary Figure 4B**).

Clinically recognizable lesions developed earlier in IMQ-treated WT mice (day 5) compared to GzmK KO mice (day 6) (**Figure 2B**). Erythema and desquamation severity were reduced in GzmK KO mice, as determined using the modified psoriasis area and severity index (PASI) scoring system (scoring outline listed in **Supplementary Table 4**) (40). Both sets of individual scores were significantly reduced in GzmK KO mice at days 6 and/or 7 (**Figure 2C**). Accordingly, GzmK KO mice exhibited a reduced cumulative severity score (defined as combined erythema and desquamation severity scores) for the duration of the study and a significant decrease in cumulative severity score at day 7 compared to WT mice (**Figure 2C**). Other phenotypes observed in GzmK KO mice included reduced inflammatory infiltrate in the upper dermis and the absence of parakeratosis and vascular dilation in the papillary dermis compared to WT mice (**Figure 2D**, **Supplementary Table 5**). Altogether, these results indicate that GzmK deficiency attenuates disease severity in the murine model of IMQ-induced psoriasis.

### 2.3 GzmK depletion reduces IMQ-induced inflammatory cell infiltrate

**Figure 3:**
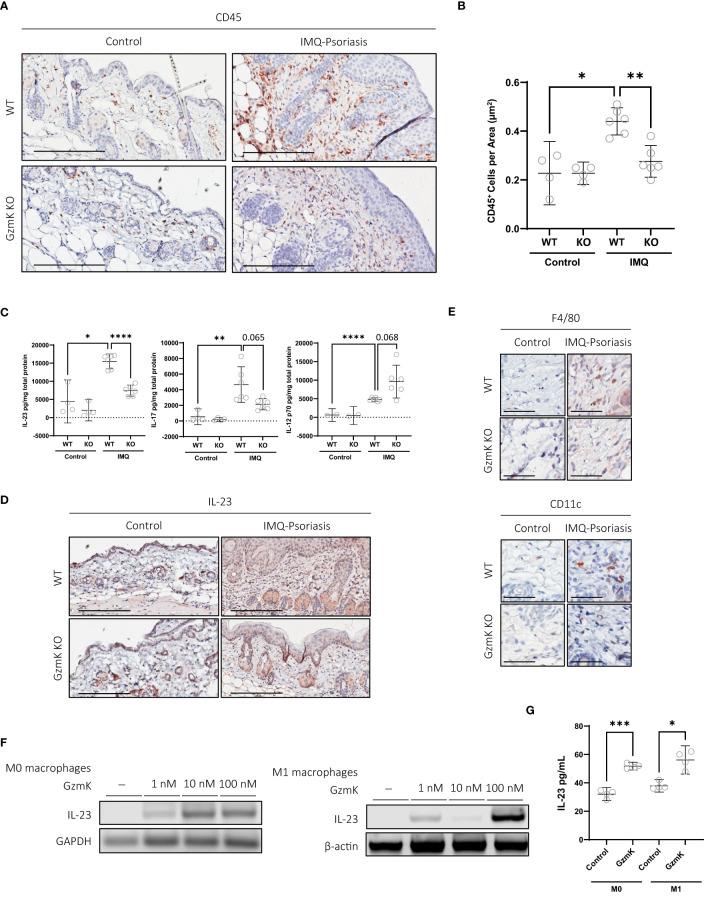
GzmK depletion reduces IMQ-induced inflammatory cell infiltrate, GzmK induces IL-23 secretion from macrophages. **(A)** CD45 immunohistochemistry of dorsal skin in untreated and IMQ-treated WT and GzmK KO mice. **(B)** Data presented as number of CD45-positive cells per area (um^2^). **(C)** Quantification of IL-12, IL-17 and IL-23 in WT and GzmK KO tissue homogenates by ELISA. Data presented as ρg cytokine per mg total protein. **(D)** IL-23A immunohistochemistry of dorsal skin in untreated and IMQ-treated WT and GzmK KO mice. **(E)** F480 (monocytes/macrophages) and CD11c (dendritic cells) immunohistochemistry of dorsal skin in untreated and IMQ-treated WT and GzmK KO mice skin. **(F)** IL-23 gene expression analyzed by RT-PCR in THP-1 macrophages ± GzmK exposure. β-actin and GAPDH were used as loading controls. **(G)** IL-23 protein secretion analyzed by ELISA of culture medium from THP-1 macrophages ± GzmK exposure. Data presented as ρg cytokine per ml. n ≥ 3 per group **(A-G)**. Data were analyzed by Brown-Forsythe and Welch ANOVA tests with Dunnett’s T3 *post-hoc* test for multiple comparisons and presented as mean with 95% CI **(B, C)**, or Welch’s t-test and Mann-Whitney test and presented as mean with 95% CI **(G)**. In all plots, ^*^ *P* ≤ 0.05, **P ≤ 0.01, ^***^ *P* ≤ 0.001, ^****^ *P* ≤ 0.0001. Scale bars represent 200 µm **(A, D)** and 50 µm **(E)**, with representative images shown.

Compared with IMQ-treated WT mice, GzmK KO mice displayed an attenuated inflammatory response, characterized by a decreased leukocyte infiltration (**Figures 3A, B**). T cells (CD3^+^) were most abundant in both IMQ-treated WT and GzmK KO mice compared to other cell types, similar to human psoriatic tissue (41). However, there was a decrease in the number of CD3^+^ per area in IMQ-treated lesions of GzmK KO mice compared to WT mice (**Supplementary Figures 5A, B**). Additionally, a decrease in microvascular density (CD31^+^) was observed in IMQ-treated GzmK KO mice compared to WT mice (**Supplementary Figures 5C, D**). These findings highlight the potential importance of GzmK in modulating inflammatory responses during disease development.

### 2.4 GzmK induces IL-23 secretion from macrophages

In IMQ-treated GzmK KO mice, IL-17 and IL-23 tissue levels were reduced compared to IMQ-treated WT mice, whereas IL-12p70 levels were increased in GzmK KO mice (**Figure 3C**). To examine the lesional tissue distribution of IL-23, immunohistochemistry was performed. IL-23A staining of murine tissues identified that the majority of IL-23^+^ cells were located in the dermis within the vicinity of blood vessels and exhibited a morphology characteristic of mononuclear cells (**Figure 3D**). In contrast to GzmK KO mice, infiltration of macrophages (F4/80^+^) (CD11c^+^), two major sources of IL-23 in psoriasis (42), were observed in IMQ-treated WT mice (**Figure 3E**). The role of GzmK in inducing IL-23 release from macrophages was therefore investigated. THP-1-derived M0 and M1 macrophages were exposed to increasing concentrations of active recombinant human GzmK. GzmK increased IL-23 mRNA expression in a dose-dependent manner and promoted IL-23 secretion from both M0 and M1 macrophages (**Figures 3F**, **G**). These results support a pro-inflammatory function for GzmK in psoriasis through the stimulation of IL-23 production.

### 2.5 GzmK depletion attenuates IMQ-induced epidermal hyperplasia

**Figure 4:**
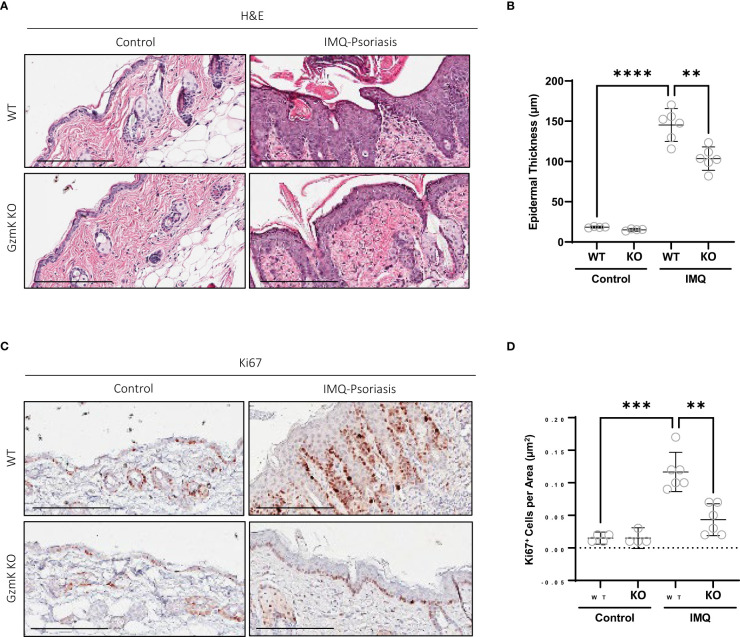
GzmK depletion attenuates IMQ-induced epidermal proliferation. **(A)** Hematoxylin and Eosin staining of dorsal skin in untreated and IMQ-treated WT and GzmK KO mice comparing epidermal thickness. **(B)** Data presented as mean epidermal thickness (µm). **(C)** Ki67 immunohistochemistry of dorsal skin in untreated and IMQ-treated WT and GzmK KO mice. **(D)** Data presented as number of Ki67-positive cells per area (µm^2^). n ≥ 6 per group **(A-D)**. Data were analyzed by Brown-Forsythe and Welch ANOVA tests with Dunnett’s T3 *post-hoc* test for multiple comparisons and presented as mean with 95% CI **(B, D)**. In all plots, **P ≤ 0.01, ^***^ *P* ≤ 0.001, ^****^ *P* ≤ 0.0001. Scale bars represent 200 µm **(A, C)**, with representative images shown.

The contribution of GzmK to epidermal hyperplasia, a hallmark of psoriasis, was assessed in the murine model of IMQ-induced psoriasis-like skin inflammation. IMQ-treated GzmK KO mice exhibited a reduction in epidermal thickness compared with WT mice at day 7 (**Figures 4A, B**). In IMQ-treated GzmK KO mice and untreated controls, the proliferation marker Ki67 was restricted to basal keratinocytes, whereas in IMQ-treated WT mice, a large number of suprabasal keratinocytes also exhibited positive staining for this marker (**Figure 4C**). Comparatively, GzmK KO mice exhibited a decrease in the mean number of Ki67^+^ cells per µm^2^ compared with WT mice (**Figure 4D**). In IMQ-treated GzmK KO mice, a similar decrease in proliferating cell nuclear antigen (PCNA) was observed compared to IMQ-treated WT (**Supplementary Figure 6A**). Further, when both primary (NHEKs) and immortalized (HaCaTs) keratinocytes were cultured in the presence of GzmK, increased levels of PCNA were observed (**Supplementary Figure 6B**). Consequently, GzmK may contribute to psoriasis epidermal hyperplasia through the stimulation of keratinocyte proliferation.

### 2.6 GzmK stimulates human keratinocyte proliferation via the PAR-1/MAPK/STAT3 pathway

**Figure 5:**
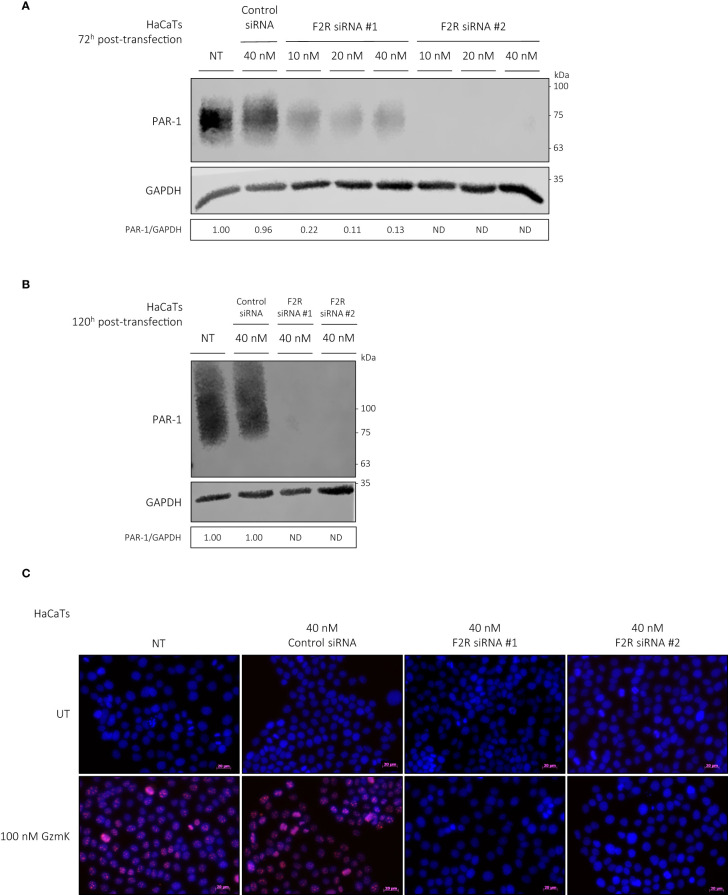
GzmK stimulates human keratinocyte proliferation via PAR-1. **(A)** Western blot immunodetection of PAR-1 and GAPDH of HaCaTs transfected with or without *F2R* (PAR-1) or Control siRNA (10 nM or 20 nM or 40 nM, 72 h). **(B)** Western blot immunodetection of PAR-1 and GAPDH of HaCaTs transfected with or without *F2R* (PAR-1) or Control siRNA (40 nM, 120 h). **(C)** HaCaTs transfected with or without *F2R* (PAR-1) or Control siRNA (40 nM, 120 h) and stimulated with or without GzmK (100 nM, 48 h). Proliferation was detected by Ki67 immunocytochemistry, Ki67 = red, Hoechst nuclear stain = blue. n ≥ 3 per group **(C)**. Scale bars represent 20 µm **(C)**, with representative images shown.

As documented previously, both normal human epidermal keratinocytes (NHEKs) and keratinocyte cell lines (HaCaTs) express PAR-1 (43, 44). GzmK is known to activate PAR-1 through cleavage of its tethered ligand, initiating receptor activation and cellular responses from various cell types (21, 24, 26). We examined whether extracellular GzmK was inducing proliferation through the cleavage and subsequent activation of PAR-1. To address this, we used 2 siRNA sequences designed against human *F2R* (PAR-1). Both siRNAs were transfected at a final concentration of 40 nM in HaCaTs, which achieved an 87% (siRNA #1) and 100% (siRNA #2) reduction at 72 h post-transfection and 100% (siRNA #1 and #2) at 120 hours post-transfection (**Figures 5A, B**). Those HaCaTs transfected with PAR-1 siRNA demonstrated a marked reduction of GzmK-mediated proliferation as visualized by Ki67 immunocytochemistry (**Figure 5C**), supporting our hypothesis that GzmK contributes to keratinocyte proliferation through a PAR-1 dependent extracellular mechanism.

As activation of the MAPK signaling pathway has been previously linked to keratinocyte proliferation (45–47), we next assessed their potential downstream activation by PAR-1 in keratinocytes. Western blot analysis revealed a robust phosphorylation of p38 (MAPK14) and p44/42 (ERK1/2), but not p46/54 (SAPK/JNK) MAPK, in keratinocytes stimulated by GzmK for up to 3 hours (**Supplementary Figures 7A–C**).

**Figure 6:**
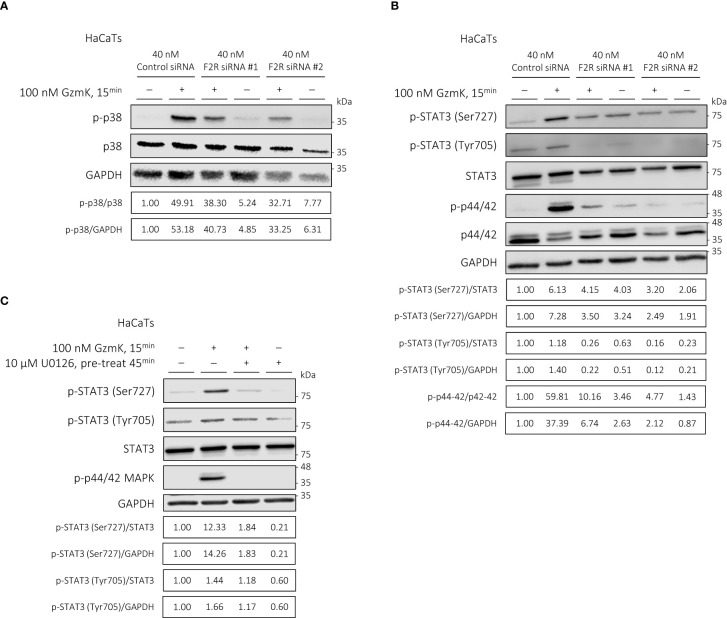
GzmK activation of PAR-1 phosphorylates p38 and p44/42 MAPK in human keratinocytes; GzmK induces STAT3 (Ser727) phosphorylation in a PAR-1/p44/42 MAPK-dependent manner in human keratinocytes. **(A)** Western blot immunodetection of p-p38 MAPK, total p38 MAPK, and GAPDH of HaCaTs transfected with or without *F2R* (PAR-1) or Control siRNA and stimulated with or without GzmK (100 nM, 15 min). **(B)** Western blot immunodetection of p-STAT3 (Ser727), p-STAT3 (Tyr705), total STAT3, p-p44/42 MAPK, total p44/42 MAPK, and GAPDH of HaCaTs transfected with or without *F2R* (PAR-1) or Control siRNA and stimulated with or without GzmK (100 nM, 15 min). **(C)** Western blot immunodetection of p-STAT3 (Ser727), p-STAT3 (Tyr705), total STAT3, p-p44/42 MAPK, and GAPDH of HaCaTs pre-treated with U0126 (10 µM, 45 min) followed by stimulation with or without GzmK (100 nM, 15 min).

We next examined the involvement of PAR-1 in GzmK-dependent MAPK signaling. When keratinocytes were transfected with *F2R* (PAR-1) siRNA as above, GzmK-mediated phosphorylation of both p38 and p44/42 MAPK was inhibited (**Figures 6A, B**). Taken together, the findings suggest that GzmK activation of the PAR-1/MAPK pathway contributes to the induction of keratinocyte proliferation.

To further understand the downstream signaling pathways involved in GzmK/PAR-1/MAPK-mediated keratinocyte proliferation, STAT3 phosphorylation was assessed. Upon GzmK stimulation of human keratinocytes, we observed a marked increase in STAT3 phosphorylation at Ser727 and to a lesser extent at Tyr705, both peaking at 15 mins and maintaining elevated levels up to 3 hours-post stimulation (**Supplementary Figure 7A**). This finding suggests a sustained activation of STAT3 signaling in response to GzmK.

We subsequently examined whether the observed GzmK-mediated STAT3 signaling was PAR-1 and/or MAPK pathway-dependent. Keratinocytes transfected with *F2R* (PAR-1) siRNA showed inhibited GzmK-mediated phosphorylation of STAT3 at both Ser727 and Tyr705 (**Figure 6B**). To determine whether this effect occurs downstream of MAPK, keratinocytes were pre-treated with U0126, a p44/42 MAPK inhibitor. Following this treatment, the phosphorylation of STAT3 Ser727 was notably abrogated (**Figure 6C**), indicating that STAT3 phosphorylation occurs downstream of PAR-1-mediated p44/42 activation.

## 3 Discussion

The mechanisms by which psoriasis is regulated are poorly understood. Currently, mechanisms underlying IL-23-driven inflammation and STAT3-driven keratinocyte proliferation are areas of high therapeutic interest and active investigation.

Of relevance to the present study, a role for serine proteases and their endogenous inhibitors (Kallikreins-Related Peptidases (48, 49), Marapsin (50), Vaspin (51), SerpinB7 (52), Leukocyte Elastase (53)) in psoriasis have been investigated. When compared with other classes of proteases, serine proteases are among the most likely to mediate psoriasis pathology since a majority are released to the extracellular milieu indiscriminately (54). The emerging pro-inflammatory effects of the serine protease, GzmK, initially prompted us to explore its potential role in psoriasis pathogenesis. Further, chromosome 5q, which contains the GzmK gene in humans, includes a susceptibility region for psoriasis (55) and numerous other autoimmune and inflammatory conditions including Crohn’s disease (56), rheumatoid arthritis (57), asthma (58), and atopic dermatitis (59).

In the current study, GzmK levels were significantly increased in skin lesions from human psoriasis patients, predominantly within the dermal inflammatory cell infiltrate. Macrophages were identified as the largest population of GzmK-positive cells present exclusively within psoriasis lesions. It is known that infiltrating macrophages are recruited in inflammatory conditions and play an important role in psoriasis development, particularly during the initiation stages (60). Further, M1 polarization in psoriasis is associated with increased disease severity (61). Hence, GzmK produced and secreted by inflammatory macrophages may contribute to early psoriasis development. This is supported by a previous study in acute thermal injury, where GzmK was reported to be expressed and secreted by inflammatory macrophages (21). Monocytes/macrophages are also the dominant immune population in the synovial fluid of psoriatic arthritis, an inflammatory spondyloarthritis that affects up to 30% of people with psoriasis (62). Although the proportion of GzmK-positive mast cells in psoriatic lesions does not significantly differ from that in healthy skin, this does not rule out a pathogenic role in psoriasis. Notably, approximately 30% of dermal mast cells in psoriatic lesions lack the endogenous inhibitor for GzmK, bikunin (63). The prevalence of bikunin-negative mast cells in healthy controls is unclear; however, in both atopic dermatitis and chronic eczema, almost all tryptase-positive mast cells also express bikunin (63). This suggests that the lack of bikunin expression in dermal mast cells may be unique to psoriasis. Despite the presence of GzmK-positive mast cells in both healthy and psoriatic skin, their potential contribution to the development of psoriasis warrants further investigation. The present study suggests that there may be multiple cell sources that contribute to elevated GzmK that is observed in psoriasis lesions. It is important to note that the pathogenic impact of GzmK observed in the current study is specifically attributed to the proteolytic activity of extracellular GzmK regardless of the independent cellular source(s).

The functional role of GzmK in psoriasis was studied in WT and GzmK KO mice using the established IMQ model of psoriasis-like skin inflammation (39). The IMQ-induced psoriasis model is amongst the most widely used mouse models of human psoriasis. It recapitulates several histological and immunological features of human psoriasis including an IL-23 and/or IL-17 dependent inflammatory response (39, 64–67). Accordingly, IMQ-treated WT mice presented with multiple features reminiscent of the phenotype observed in human psoriasis lesions including increased epidermal thickness/hyperplasia, perivascular inflammation (predominantly characterized by the presence of CD3^+^ T cells), vascularization, and secretion of IL-23/IL-17 axis cytokines. GzmK contributed to disease severity in the murine model of IMQ-induced psoriasis, as reflected in earlier clinical disease with more severe erythema and desquamation in WT mice compared to GzmK KO mice. Histologically, GzmK primarily contributed to disease severity by inciting lymphocytic infiltration and epidermal hyperplasia.

Of particular relevance to clinically approved biologics, GzmK depletion in GzmK KO mice reduced the levels of both IL-17 and IL-23 in IMQ-induced psoriasis-like lesional skin. The decreased inflammatory reaction associated with the lower production of these cytokines provides insights with respect to the milder disease phenotype observed in GzmK KO mice compared to WT mice. These findings suggest that GzmK contributes to a pro-inflammatory response that favours the Th17 lineage and is upstream of IL-23/IL-17. In support, GzmK induced macrophage production and secretion of IL-23. IL-23 is a heterodimer composed of two subunits: p19 and p40, which it shares with IL-12. For this reason, previous therapies targeting IL-23p40 have also targeted IL-12. However, IL-12 is important for the activation of Th1 cells and recent research has revealed that IL-12 may serve a protective role in psoriasis (68, 69). Interestingly, IL-12 levels were heightened in GzmK KO mice lesional skin compared to WT mice, which may also contribute to the milder phenotype observed. Thus, by inhibiting GzmK, it would appear we are able to inhibit IL-23 and not IL-12, bringing several advantages over current therapies. Further, there is strong evidence to support the role of IL-17 in the protection of epithelial barrier function in the gut mucosa and airways; as such, systemic inhibition of IL-17 presents potential risk of candida infection and is associated with infection and disease worsening in these tissues (70–73). Although IL-23 is upstream of IL-17, studies suggest that blocking IL-23 does not impair IL-17 production by innate non-T-cell lymphocytes in the gut (74), thereby preserving intestinal integrity (72, 75). Provided the role of GzmK in selectively inducing IL-23p19, it could be a potential therapeutic target for psoriasis treatment.

Further, IL-23 binds to the IL-23 receptor (IL-23R) which, at least in Th17 cells, is induced by stimulation with IL-1β, IL-6 and transforming growth factor (TGF)-β. Previously, we have demonstrated that GzmK can induce macrophages and resident skin cells to secrete IL-1β and IL-6 (21), emphasizing its potential support of IL-23/IL-17 pathogenic mechanisms. Together, the overproduction of pro-inflammatory cytokines, IL-1β, IL-6, IL-17, and IL-23, associated with elevated GzmK would be expected to elicit a severe inflammatory imbalance thereby driving keratinocyte proliferation and psoriatic plaque development.

In addition, this phenotype is exacerbated by the ability of GzmK to directly induce keratinocyte proliferation and epidermal hyperplasia. Previous research has demonstrated that GzmK can induce PAR-1-mediated proliferation in human fetal lung fibroblasts (24). Extending these findings, our study reveals that GzmK induces PAR-1-mediated proliferation in keratinocytes. Furthermore, we observed that GzmK concurrently activates p38 and p44/42 MAPK pathways, with the latter specifically leading to the phosphorylation of STAT3 (predominantly at Ser727). In addition to STAT3, the significance of PAR-1 and MAPK signaling in psoriasis is well-established with respect to keratinocyte proliferation (45–47). Within psoriasis lesions, PAR-1, p38, and p44/42 MAPK are consistently active and present throughout the epidermis (48, 76). Additionally, the modulation of STAT3 transcriptional activity in keratinocytes by MAPK signaling has been observed (77). Provided the interconnected nature of these signaling events, our findings underscore the therapeutic potential of targeting GzmK, not only as a means to modulate IL-23-driven inflammation, but also to regulate STAT3-driven keratinocyte proliferation through the PAR-1/MAPK pathway, offering a novel approach to psoriasis management. A schematic overview depicting the role of GzmK in psoriasis can be seen in the Graphical Abstract.

At present, pharmacologic agents targeting GzmK activity do not exist. However, there is a natural physiological inhibitor of human and mouse GzmK found in plasma, known as Inter-alpha inhibitor protein (IaIP). IaIP acts as an extracellular inhibitor, mediated by the second Kunitz-type domain of its bikunin subunit (78). In a survey of over 2,007 genes expressed in epithelial tissues, Itoh et al. identified 7 genes that show high expression levels only in non-lesional/uninvolved psoriasis skin compared to matched lesional psoriasis skin, which includes *AMBP*, the precursor for alpha-1-microglobulin and bikunin (79). In this case, free GzmK may be accumulating and show upregulated activity in lesional psoriasis skin.

Although several treatment interventions for psoriasis exist, barriers to effective care include cost of advanced biologics, cumulative toxicity, and potential for adverse events (5, 6, 80). As GzmK is a protease and produced locally in affected tissues, the present study offers an alternative approach that could mitigate expensive biological approaches and associated long-term off-target effects.

## 4 Materials and methods

### 4.1 Human database queries

Gene Expression Omnibus (GEO) (https://www.ncbi.nlm.nih.gov/geo/) was used to retrieve raw scRNA-seq gene expression profiles from healthy control subjects, non-lesional and lesional psoriasis patients (accession numbers: GSE13355, GSE30999). The GSE13355 dataset (GDS4602) was generated using human skin punch biopsies obtained from 58 psoriasis patients that sampled both lesional and non-lesional skin and 64 healthy control subjects (33–35), while the GSE30999 dataset (GDS4600) included lesional skin collected from 85 psoriasis patients along with matched biopsies of non-lesional psoriasis skin (36, 37). The resulting datasets were queried for *GZMK* normalized gene expression using the GEO Profiles database. The expression values in the table are after adjustment of RMA expression values (on the log scale) to account for batch and sex effects as appropriate.

SEEK (http://seek.princeton.edu/) is a search engine used to identify/query gene(s) co-expression with other genes or datasets. After defining the search range to (non-cancer) skin-related datasets, available datasets were ordered by their relevance to/enrichment for *GZMK* (see **Supplementary Tables 1, 2**).

### 4.2 Human samples

Paraffin-embedded psoriasis skin was obtained retroactively from patients diagnosed with psoriasis (*N* > 3). Psoriasis patients had not received any treatments prior to biopsy (additional patient data is available in **Supplementary Table 3**). Paraffin-embedded healthy control skin was obtained from subjects undergoing elective abdominoplasty (*N* ≥ 3).

### 4.3 Mice

All animal ethics and procedures were approved and performed in accordance with the guidelines for animal experimentation as per the University of British Columbia (UBC) Animal Care Committee. C57BL/6 mice (listed as WT in text) were purchased from Jackson Laboratories (Bar Harbor, ME). GzmK knockout mice (C57BL/6 background) (listed as GzmK KO in text) were kindly gifted from Dr. Phillip Bird (Department of Biochemistry and Molecular Biology, Monash University, Australia) and are routinely backcrossed with C57BL/6 mice. Both WT and GzmK KO mice were bred and housed at the International Collaboration on Repair Discoveries (ICORD) vivarium. Six female mice (aged 6-8 weeks) were used per genotype.

### 4.4 IMQ-induced psoriasis model

The IMQ-induced mouse model of psoriasis was performed as previously described (39). Daily topical administration of IMQ on shaved dorsal skin induced erythematous, scaly lesions resembling human psoriasis. On day 8 of the experiment, mice were euthanized, and tissue collected for analysis.

### 4.5 Immunostaining & histology

Five-micrometer-thick, vertical sections were prepared from all formalin-fixed, paraffin-embedded skin samples and processed for staining using Hematoxylin and Eosin for morphological analysis, Toluidine Blue O (TBO) for mast cells, or specific antibodies for immunohistology as listed in **Supplementary Table 6**. All histological and histochemical stains were imaged with the Aperio CS2 slide scanner (Leica Biosystems). The resulting images were prepared using the Aperio ImageScope Viewer (Leica Biosystems) or QuPath (University of Edinburgh) (81).

The density of positive cells and/or intensity of staining was determined using the formula: (number of positive cells or total intensity of strong positive staining)/(total skin area examined), which was measured using the Aperio ImageScope Viewer (Leica Biosystems) or by evaluating H-Score or Positive Cell Detection using QuPath (University of Edinburgh). Epidermal thickness was determined using the formula: (tissue epidermal area)/(tissue length), which was measured using the Aperio ImageScope Viewer (Leica Biosystems). Areas of skin layers (dermis and subcutaneous fat), hair follicles and glands were carefully excluded as appropriate.

### 4.6 Mice scoring of disease severity (modified psoriasis area and severity index)

Evaluations of disease severity were performed daily using a modified version of the Psoriasis Area and Severity Index as previously described (**Supplementary Table 4**) (40). IMQ-induced psoriasis-like skin severity was graded independently by two dermatology researchers. The two scores were averaged.

### 4.7 Tissue sampling

Biopsies from the dorsal region were harvested and placed in formalin for subsequent paraffin-embedding or snap-frozen in liquid nitrogen.

### 4.8 Primary and immortalized cells

Human keratinocytes (HaCaTs) were cultured in DMEM (Sigma-Aldrich) containing 10% (volume/volume) fetal bovine serum and 1% (volume/volume) penicillin/streptomycin. Normal human keratinocytes (NHEKs) were cultured in Keratinocyte Basal Medium 2 with supplements (Promo Cell) and 1% (volume/volume) penicillin/streptomycin. Human THP-1 monocytes (ATCC^®^ TIB202™) were cultured and differentiated into M0 macrophages, and polarized into M1 macrophages, as previously described (82). Cellular phenotypes of cells transformed from THP-1 monocytes were validated using flow cytometry (**Supplementary Figure S8A**). All cells were cultured in serum-free media conditions for 24 hours before and during each experiment. Where pertinent, cells were treated with rhGzmK protein (BonOpus, non-commercial supply).

### 4.9 siRNA transfection

HaCaT cells were seeded into 6-well plates at a density of 0.25 x10^6^ cells/well approximately 24 h before transfection. Two siRNA targeting the *F2R* gene (PAR-1) and a negative control siRNA with no complementary target sequences were used (Qiagen). Cells were transfected with 10-40 nM siRNA using HiPerFect Transfection Reagent (Qiagen), as per supplier instructions. The efficiency of gene knockdown was evaluated by Western blot after incubation in normal cell culture conditions for 72 and 120 h.

### 4.10 Statistics

Coefficient of Variation (CV) was equal to an average of 1%. CV ≤ 5% was defined a-priori as the acceptable limit. Data was analyzed using Graphpad Software for Windows. Data was analyzed for normal (Gaussian) distribution using the Shapiro-Wilk test (alpha=0.05). Statistical significance was determined by t-tests or ANOVA with *post-hoc* test for multiple comparisons, as appropriate (particulars of statistical tests specified in figure legends). ^*^ *P* ≤ 0.05 was defined as statistically significant.

### 4.11 Ethics statement

This study was carried out in accordance with the recommendations of institutional guidelines of the University of British Columbia. All human studies were approved by the University of British Columbia Human Research Ethics Committee. All animal experiments were approved by the Animal Care Committee.

## Data availability statement

The original contributions presented in the study are included in the article/**Supplementary Material**. Further inquiries can be directed to the corresponding author.

## Ethics statement

The studies involving humans were approved by University of British Columbia Clinical Research Ethics Board. The studies were conducted in accordance with the local legislation and institutional requirements. Written informed consent for participation was not required from the participants or the participants’ legal guardians/next of kin because the samples used were excess tissue slides that were generated for diagnostic purposes and all samples were fully de-identified. The animal study was approved by University of British Columbia Animal Care Committee. The study was conducted in accordance with the local legislation and institutional requirements.

## Author contributions

KR: Conceptualization, Formal analysis, Funding acquisition, Investigation, Methodology, Project administration, Resources, Visualization, Writing – original draft, Writing – review & editing. AA: Investigation, Methodology, Writing – review & editing. CT: Methodology, Writing – review & editing. LN: Methodology, Writing – review & editing. SH: Methodology, Writing – review & editing. MP: Investigation, Writing – review & editing. RC: Investigation, Writing – review & editing. HZ: Resources, Writing – review & editing. KJ: Funding acquisition, Writing – review & editing. AB: Investigation, Writing – review & editing. RC: Resources, Writing – review & editing. DG: Funding acquisition, Resources, Writing – review & editing.
