# Supplementary material for: Granzyme K mediates IL-23-dependent inflammation and keratinocyte proliferation in psoriasis
Source: Front Immunol. 2024 Jun 5;15:1398120. doi: 10.3389/fimmu.2024.1398120 (PMC11188347; doi:10.3389/fimmu.2024.1398120)
Supplement: Supplementary file 1 [file DataSheet_1.pdf]

Supplementary Material

Granzyme K Mediates IL-23-Dependent Inflammation and Keratinocyte Proliferation in Psoriasis

Katlyn C. Richardson, Alexandre Aubert, Christopher T. Turner, Layla Nabai, Sho Hiroyasu, Megan A. Pawluk, Rachel A. Cederberg, Hongyan Zhao, Karen Jung, Angela Burleigh, Richard I. Crawford, and David J. Granville*

*** Correspondence:** David J. Granville: dgranville@icord.org

1 Supplementary Materials & Methods

1.1 Sequence Alignment and Homology

Sequence alignment and identification of homology degree between human (according to the Uniprot accession no. P49863) and mouse (according to the Uniprot accession no. O35205) Granzyme K has been performed using the CLUSTAL Omega software (http://www.clustal.org/omega/). Residues labelled with (*) are identical between the two granzymes, residues labelled with (:) are highly conserved and residues labelled with (.) are poorly conserved. Amino-acids from the catalytic triad (His^{57}-Asp^{102}-Ser^{195}, according to Hink-Schauer et al., 2002) are identified in bold.

1.2 Flow Cytometry

Adherent THP-1-derived M0 and M1 macrophages were harvested using Cell Dissociation Buffer (Gibco). All cells were harvested, washed and resuspended in Hanks' balanced salt solution with 10 mM HEPES (StemCell Technologies) + 2% FBS + 0.05% NaN_3. Anti-human CD16/32 (eBioscience) was used to block cells prior to antibody staining. Cells were stained with the following antibodies on ice for 30 minutes: anti-human CD14-FITC (367115), anti-human HLA-DR APC (307609), anti-human CD68-PE (333807), anti-human CD206-APC-Cy7 (321119) (all Biolegend) (listed in **Supplementary Table 6**). Cells were then washed and resuspended in viability dye Propidium Iodide PE-Texas Red (Biolegend). All samples were acquired on a BD LSRFortessa (FACSDiva Software, BD) and analyzed with FlowJo software platform (TreeStar Inc.).

1.3 Reverse Transcriptase Polymerase Chain Reaction (RT-PCR)

RNA was isolated using the RNeasy Mini Kit (Qiagen) according to the manufacturer's instructions. cDNA was generated using the First strand cDNA Synthesis Kit (Origene, NP100042) according to the manufacturer's instructions. Human IL-23 was amplified using custom primers (Invitrogen). Thermocycling was as follows: 95 °C, 2 minutes; then 42 cycles of 95 °C, 30 seconds, 55 °C, 30 seconds, and 72°C, 30 seconds; then 72 °C, 5 minutes. Amplification of β-actin or GAPDH was used as control. PCR products were separated on a 2% agarose gel and visualized using a LI-COR Odyssey Fc system (Lincoln) under the 600-nm channel.

1.4 Western Blotting

Total proteins were extracted by cell scraping and homogenization using 1X RIPA extraction buffer containing protease inhibitor and phosphatase inhibitor cocktails, followed by incubation for 20 min on ice. Following, cells were centrifuged at 12,500 rpm for 10 min at 4°C, and supernatant containing solubilized proteins was collected and stored at -20°C.

20 µg of total proteins were resolved by SDS-PAGE in Tris-Glycine (TG) Buffer containing 0.1% SDS and then transferred onto Polyvinylidene Fluoride (PVDF) membrane (Millipore, PVH00010) at 0.4 Amps for 2 h in TG-SDS buffer containing 10% Ethanol. Membranes were blocked with 5% or 10% (w/v) non-fat dry milk diluted in Tris-buffered saline containing 0.1% (v/v) Tween-20 (TBS-T) for 1 h at room temperature followed by 3x10 min washes in TBS-T and overnight incubation with primary antibodies (listed in **Supplementary Table 6**) at 4°C. The next day, membranes were washed 3x10 min in TBS-T followed by incubated with secondary antibodies, anti-mouse IgG or anti-rabbit IgG HRP (diluted at 1:10,000 in TBS-T containing 5% or 10% (w/v) non-fat dry milk) for 1 h at room temperature. Membranes were subsequently washed 3x10 min in TBS-T. Signal was detected using the Pierce ECL Western Blotting Substrate (Thermo, 32106) or SuperSignal West Pico PLUS Chemiluminescent Substrate (Thermo, 34577) and visualized using a LI-COR Odyssey Fc system (Lincoln) under the 700-nm (0.5 min) and Chemi (2 min) channels. In the instance that membranes were probed with phosphorylated MAPK antibodies (p-p38, p-p44/42, p-p46/54), membranes were neutralized with 0.02% sodium azide (1x 30 min) prior to re-incubation with antibodies targeting the total forms (p38, p44/42, p46/54). In the instance that membranes were probed with phosphorylated STAT3, GzmK or PCNA antibodies, membranes were stripped with 0.2 M NaOH (minimum 2x 5 min) prior to re-incubation with antibodies targeting another phosphorylation site (STAT3), the total form (STAT3), or loading control (GAPDH for GzmK or PCNA). Signal readouts were analyzed with Fiji software platform (ImageJ).

1.5 Enzyme-Linked Immunosorbent Assays (ELISA)

Lesioned dorsal skin was sampled from each mouse and flash frozen with liquid nitrogen and stored at -80°C until use. The samples were homogenized with mortar and pestle plus liquid nitrogen and lysed with a combination of RIPA lysis buffer (0.1% SDS, 150nM NaCl, 50nM Tris, 0.5% Sodium deoxycholate, 1.0% Triton X-100) plus protease inhibitor cocktail, and sonification (Qsonica, Q125) (3x10 seconds, 50% intensity, 1 minute rest between). Samples were then centrifuged at 18,000 x g for 10 min. and the supernatants collected, aliquoted and stored at −80°C. The total protein concentrations in the lysates were quantified using the Pierce™ BCA Protein Assay Kit (Thermo Scientific, 23225), according to the user's manual. Mouse IL-12p70 (M1270), IL-17 (M1700) and IL-23 (M2300) ELISA kits (all R&D Systems) were used according to the manufacturer’s instructions.

Cell culture supernatants were collected and centrifuged at 10,000xg for 10 min. and the supernatants collected, aliquoted and stored at -20°C. Human IL-23 (R&D Systems, D2300B) was used according to the manufacturer’s instructions.

1.6 Immunocytochemistry

HaCaTs, cultured on a 6 well-plate, were washed with PBS, fixed with 4% paraformaldehyde and permeabilized with Triton X-100. Cells were incubated with 1% BSA in 0.1% PBS-Tween 20, then incubated with a primary antibody against Ki67 in block. The cells were subsequently incubated with AlexaFluor 594-donkey anti-rabbit IgG (1:700) for 90 min. The nuclei of the cells were stained with

Hoechst 32224 (1:5000) for 1 min. The slides were observed on an epifluorescent microscope under Texas Red and DAPI channels at 10X and 20X magnification.

2 Supplementary Figures Legends

Supplementary Figure 1. Extended data on GzmK cellular co-localization in healthy skin and lesional psoriasis – focus on mast cells

(A) Quantification of GzmK staining in the extracellular milieu presented as extracellular GzmK staining intensity per area (μm^2) in the skin of healthy control subjects and psoriasis patients. (B) Sequential immunostaining of TBO and GzmK in the skin of healthy control subjects and psoriasis patients. Red arrows indicate co-staining cells (GzmK$^+$/TBO$^+$). Green arrows indicate non-co-staining cells (GzmK$^+$/TBO$^-$). (C) Quantification of GzmK-positive cells that are also TBO-positive presented as GzmK-positive cells that are TBO-positive (%) in the skin of healthy control subjects and psoriasis patients. (D) Two-colour immunofluorescence of GzmK and tryptase in the skin of healthy control subjects and psoriasis patients. $N \geq 3$ per group (A-D). Data were analyzed by Welch's t-test and presented as mean with 95% CI (A, C). In all plots, $^{*}P \leq 0.05$, $^{**}P \leq 0.01$, $^{***}P \leq 0.001$, $^{****}P \leq 0.0001$. Scale bars represent 200 μm (B), with representative images shown.

Supplementary Figure 2. Extended data on GzmK cellular co-localization in lesional psoriasis – serial staining

(A) Serial immunohistochemistry of GzmK and cell markers (various) in the skin of psoriasis patients. $N \geq 3$ per group. Scale bars represent 100 μm (A), with representative images shown.

Supplementary Figure 3. Extended data on GzmK cellular co-localization in lesional psoriasis – immunofluorescence staining

(A) Two-colour immunofluorescence of GzmK and cell markers (various) in the skin of psoriasis patients. $N \geq 3$ per group.

Supplementary Figure 4. Extended data related to IMQ-psoriasis model – design

(A) Sequence alignment for human and murine GzmK with catalytic triad and percent sequence homology shown. (B) Daily weight change in IMQ-treated WT and GzmK KO mice. $N \geq 6$ per genotype (B). Data presented as mean ± 95% CI (B).

Supplementary Figure 5. Extended data related to IMQ-Psoriasis model – inflammation

(A) CD3 (T cell) immunohistochemistry of dorsal skin in untreated and IMQ-treated WT and GzmK KO mice skin. (B) Data presented as number of CD3-positive cells per area (mm^2) and as percentage of CD3-positive cells to total cells (%) (C) CD31 immunohistochemistry of dorsal skin in untreated and IMQ-treated WT and GzmK KO mice. (D) Data presented as number of positive CD31-postive cells per area (mm^2). $N \geq 6$ per group (A-D). Data were analyzed by Brown-Forsythe and Welch ANOVA tests with Dunnett's T3 post-hoc test for multiple comparisons and presented as mean with 95% CI (B, D). In all plots, $^{*}P \leq 0.05$, $^{**}P \leq 0.01$, $^{***}P \leq 0.001$, $^{****}P \leq 0.0001$. Scale bars represent 50 μm (A) and 300 μm (C), with representative images shown.

Supplementary Figure 6. Extended data related to IMQ-Psoriasis model – proliferation

(A) PCNA immunohistochemistry of dorsal skin in untreated and IMQ-treated WT and GzmK KO mice. (B) Western blot immunodetection of PCNA and GAPDH of HaCaTs and NHEKs stimulated with or without GzmK (100 nM, 24 h). Scale bars represent 200 µm (A), with representative images shown.

Supplementary Figure 7. Extended data on MAPK and STAT3 Activation

(A) Western blot immunodetection of p-p38 MAPK, total p38 MAPK, p-STAT3 (Ser727), p-STAT3 (Tyr705), total STAT3, and GAPDH of HaCaTs stimulated with or without GzmK (100 nM, 10 min – 3 h). (B) Western blot immunodetection of p-p44/42 MAPK, total p44/42 MAPK, and GAPDH of HaCaTs stimulated with or without GzmK (100 nM, 10 min – 3 h). (C) Western blot immunodetection of p-p46/54 MAPK, total p46/54 MAPK, and GAPDH of HaCaTs stimulated with or without GzmK (100 nM, 10 min – 3 h).

Supplementary Figure 8. Extended data on THP-1-derived monocyte and macrophage phenotype

(A) Flow cytometric analysis and quantification of CD14+, CD68+, CD206+, HLA-DR+, THP-1 monocytes, M0 and M1 macrophages. Includes images of various cell types with representative flow cytometry histograms.

3 Supplementary Tables

3.1 Supplementary Table 1. SEEK database query – gene enrichment for *GZMK* in skin (non-cancer)

Rank	Dataset	Co-expression Score	Co-expression p-value	Description
1	GSE28914.GPL570	0.800005	0.00E+00	Human Skin Transcriptome during Epidermal Wound Healing
2	GSE13355.GPL570	0.74302	0.00E+00	Gene expression data of skin from psoriatic patients and normal controls
3	GSE26866.GPL571	0.638633	0.00E+00	Combined Use of Laser Capture Microdissection and Microarray Analysis Identifies Locally Expressed Disease-Related Genes in Focal Regions of Psoriasis Vulgaris Skin Lesions
4	GSE34248.GPL570	0.635188	0.00E+00	Gene expression profiling in psoriatic lesional and non-lesional skin [Set 1]
5	GSE17763.GPL570	0.634602	0.00E+00	Divergence of macrophage phagocytic and antimicrobial programs in leprosy
6	GSE36842.GPL570	0.628133	0.00E+00	Progressive Activation of Th2/Th22 characterizes acute and chronic atopic dermatitis
7	GSE11903.GPL571	0.611798	0.00E+00	Effective treatment of psoriasis with etanercept is linked to suppression of IL17 signaling, not immediate response TNF
8	GSE41662.GPL570	0.609001	0.00E+00	Gene expression profiling in psoriatic lesional and non-lesional skin [Set 2]
9	GSE41663.GPL570	0.5816	0.00E+00	Re-analysis by microarray using cDNA target of samples from psoriasis patients enrolled in an etanercept trial

10	GSE11622.GPL570	0.544989	0.00E+00	Molecular Analysis of the Vaginal Response to Estrogens in the Ovariectomized Rat and Postmenopausal Woman
11	GSE41078.GPL571	0.536817	0.00E+00	Gene Profiling of Narrow-band UVB-induced Skin Injury Defines Cellular and Molecular Innate Immune Responses
12	GSE17549.GPL570	0.531311	0.00E+00	Loss-of-function mutations in REP-1 affect intracellular vesicle transport in fibroblasts and monocytes of CHM patients
13	GSE14905.GPL570	0.509016	0.00E+00	Type I Interferon: Potential Therapeutic Target for Psoriasis?
14	GSE32924.GPL570	0.498933	0.00E+00	Nonlesional atopic dermatitis skin is characterized by broad terminal differentiation defects and variable immune abnormalities
15	GSE33169.GPL570	0.497395	0.00E+00	Gene expression profiling of negative-pressure-treated split-thickness skin graft donor site wounds reveals novel effects on epithelialization
16	GSE42305.GPL571	0.451821	0.00E+00	TREM-1 is a novel therapeutic target in Psoriasis
17	GSE27887.GPL570	0.450233	0.00E+00	Reversal of Atopic Dermatitis with Narrow-Band UVB Phototherapy and Biomarkers for Therapeutic Response
18	GSE28315.GPL6244	0.434898	0.00E+00	Gene expression pattern of skin biopsies of epidermolysis bullosa simplex patients in comparison with control subjects
19	GSE46239.GPL570	0.388113	0.00E+00	Gene Expression Analysis of Dermatomyositis Skin
20	GSE16129.GPL96	0.38273	0.00E+00	Enhanced Monocyte Response & Decreased Central Memory T Cells in Children with Invasive Staphylococcus aureus Infections
21	GSE16161.GPL570	0.379451	0.00E+00	Broad defects in epidermal cornification in atopic dermatitis (AD) identified through genomic analysis

22	GSE39477.GPL4133	0.369804	0.00E+00	Prospective transcriptomic identification and validation of a circulating biomarker panel for human lymphatic vascular insufficiency
23	GSE32473.GPL570	0.330947	0.00E+00	Gene expression is differently affected by pimecrolimus and betamethasone in lesional skin of atopic dermatitis.
24	GSE18686.GPL6947	0.29189	0.00E+00	Subpopulations of CD163 positive macrophages are classically activated in psoriasis
25	GSE18206.GPL570	0.273815	0.00E+00	Analysis of human in vivo irritated epidermis: differential profiles induced by sodium lauryl sulphate and nonanoic acid
26	GSE15101.GPL570	0.254151	0.00E+00	Extraction of high-quality epidermal RNA after NH4SCN induced dermo-epidermal separation of 4 mm human skin biopsies
27	GSE36169.GPL96	0.24487	0.00E+00	Prostaglandin D2 Inhibits Hair Growth and Is Elevated in Bald Scalp of Men with Androgenetic Alopecia
28	GSE30355.GPL570	0.221375	0.00E+00	Human keratinocytes have a response to injury that upregulates CCL20 and other genes linking innate and adaptive immunity
29	GSE11792.GPL571	0.197308	0.00E+00	Human Skin: Before and 8 weeks after Isotretinoin Treatment
30	GSE17539.GPL570	0.193357	0.00E+00	Expression profile of grafted human engineered skin substitutes compared with intact human
31	GSE32407.GPL571	0.192514	0.00E+00	A single intradermal injection of IFN-γ induces a psoriasis-like state in both non-lesional psoriatic and healthy skin
32	GSE30119.GPL6947	0.182455	0.00E+00	Genome-wide analysis of whole blood transcriptional response to community-acquired Staphylococcus aureus infection in vivo

33	GSE16161.GPL571	0.172768	0.00E+00	Broad defects in epidermal cornification in atopic dermatitis (AD) identified through genomic analysis
34	GSE16395.GPL570	0.159692	0.00E+00	Cell-Specific Gene Expression in Langerhans Cell Histiocytosis
35	GSE37157.GPL570	0.157931	0.00E+00	GENE-EXPRESSION ANALYSIS RELATED TO OLIVE POLLEN ALLERGY
36	GSE21429.GPL6480	0.156623	0.00E+00	Regulation of human skin pigmentation in situ by repetitive UV exposure - Molecular characterization of responses to UVA, UVB and SSR
37	GSE35340.GPL570	0.1461	0.00E+00	Notch is active in Langerhans Cell Histiocytosis and confers pathognomonic features on dendritic cells.
38	GSE8056.GPL570	0.14322	0.00E+00	Gene Expression Profiles in Thermally Injured Human Skin: A Temporal Microarray Analysis
39	GSE31652.GPL571	0.105967	0.00E+00	IL-17A is an essential cytokine to sustain pathogenic cell activation and inflammatory gene circuits in psoriasis vulgaris
40	GSE30768.GPL571	0.085265	0.00E+00	Post-therapeutic relapse of psoriasis associated with CD11a blockade is associated with T cells and inflammatory myeloid DCs
41	GSE23807.GPL4133	0.078509	0.00E+00	Biological response to 2.5 Gy of X-rays in 3-dimensional skin model, Epi-200.
42	GSE16357.GPL570	0.07747	0.00E+00	Effects of HHV8 infection in lymphatic endothelial cells
43	GSE9762.GPL570	0.069761	0.00E+00	Trisomy 21 fibroblasts (pevsn-affy-human-512801)
44	GSE32887.GPL570	0.068133	0.00E+00	Molecular profiling and gene expression analysis in cutaneous sarcoidosis (CS)

45	GSE41055.GPL5175	0.065641	0.00E+00	A predictive signature gene set for discriminating active from latent TB in Warao Amerindian children
46	GSE32527.GPL6244	0.062846	0.00E+00	DNA microarrays of Turner Syndrome induced pluripotent stem cells
47	GSE15101.GPL571	0.053173	0.00E+00	Extraction of high-quality epidermal RNA after NH4SCN induced dermo-epidermal separation of 4 mm human skin biopsies
48	GSE21413.GPL571	0.050739	0.00E+00	Expression profile of proliferating and differentiating primary human keratinocytes in vitro
49	GSE27864.GPL6244	0.048649	0.00E+00	Homeostatic tissue responses in skin biopsies from NOMID patients with constitutive overproduction of IL-1b.
50	GSE42114.GPL570	0.040809	0.00E+00	Creation of differentiation-specific genomic maps of human epidermis through laser capture microdissection

3.2 **Supplementary Table 2.** SEEK database query –dataset enrichment for *GZMK* in skin (non-cancer)

Term	p-value	q-value	T	A	T&A
Skin	6.3E-155	0.00E+00	151	100	100
Epidermis	1.48E-27	1.85E-25	37	100	23
Psoriasis	1.40E-24	1.16E-22	23	100	18
Keratinocytes	1.92E-16	1.20E-14	64	100	20
Dermis	3.07E-15	1.53E-13	18	100	12
Fibroblasts	2.14E-12	8.94E-11	211	100	28
Skin Diseases	1.68E-11	5.99E-10	10	100	8
Dendritic Cells	4.09E-10	1.28E-08	48	100	13
Dendrites	7.17E-10	1.99E-08	50	100	13
Foreskin	2.11E-06	5.27E-05	42	100	9
Hyperplasia	1.46E-05	3.32E-04	20	100	6
Inflammation	1.14E-04	2.38E-03	154	100	14
Neutrophils	1.03E-03	1.99E-02	41	100	6
Induced Pluripotent Stem Cells	2.99E-03	5.33E-02	67	100	7

Pluripotent Stem Cells	1.00E-02	1.67E-01	64	100	6
Extracellular Matrix	1.31E-02	2.04E-01	49	100	5
Blindness	1.42E-02	2.09E-01	18	100	3
Myeloid Cells	1.65E-02	2.18E-01	19	100	3
Pneumonia	1.65E-02	2.18E-01	19	100	3

p-value, p-value adjusted for multiple hypothesis testing (Benjamini-Hochberg); q-value, minimum false discovery rate; T, term size; A, number of datasets retrieved with annotations in database; T&A, size of overlap between the retrieved datasets and the annotation database

3.3 **Supplementary Table 3.** Psoriasis patient data

Patient ID	Clinical Dx	Evaluator	Pathologic Dx	Biopsy Site	Age/Sex
Psoriasis-1	query lichen planus, query psoriasis, query pityriasis chronica	Dermatologist	Psoriasis	Left upper back	69/M
Psoriasis-2	pityriasis rosea versus psoriasis versus mononucleosis after penicillin course	Family Doctor	Psoriasis	Right lower abdomen	27/M
Psoriasis-3	query guttate psoriasis, query pityriasis lichenoides chronica, query other papulosquamous condition	Dermatologist	Psoriasis	Right posterior shoulder	33/F
Healthy-1	-	-	-	Abdomen	70/F
Healthy-2	-	-	-	Trunk	21/M
Healthy-3	-	-	-	Buttock	25/F

3.4 **Supplementary Table 4.** Modified PASI scoring system

Severity Score	Erythema	Desquamation
0	None	None
1	Mild	Mild
2	Moderate	Moderate
3	Severe	Severe
4	Very Severe	Very Severe

3.5 **Supplementary Table 5.** Baker's scoring system

Skin Layer	Item	
Keratin	Munro abscess	
	Hyperkeratosis	
	Parakeratosis	
Epidermis	Thinning above papillae	
	Lengthening and clubbing of rete ridges	
	Acanthosis	
	Lack of granular layer	
Dermis	Lymphocytic infiltrate	Mild
		Moderate
		Marked
	Papillary congestion	

3.6 **Supplementary Table 6:** Key Resources

REAGENT or RESOURCE	SOURCE	IDENTIFIER
Antibodies		
mouse monoclonal anti-β-tubulin (clone AA2)	Sigma-Aldrich	Cat#05-661<br>RRID: AB_309885
rabbit monoclonal anti-CD1a (clone EP3622)	Abcam	Cat#ab108309<br>RRID: AB_10864235
mouse monoclonal anti-CD3 (clone PS1)	Abcam	Cat#ab699<br>RRID: AB_305686
rabbit polyclonal anti-CD3	Abcam	Cat#ab5690<br>RRID: AB_305055
mouse monoclonal anti-CD4 (clone 4B12)	Agilent	Cat#M7310<br>RRID: AB_2728838
mouse monoclonal anti-CD8 (clone C8/144B)	Agilent	Cat#M7103<br>RRID: AB_2075537
rabbit monoclonal anti-CD11c (clone D1V9Y)	Cell Signaling Technology	Cat#97585<br>RRID: AB_2800282
mouse monoclonal anti-CD14-FITC (clone 63D3)	Biolegend	Cat#367115<br>RRID: AB_2571928
goat polyclonal anti-CD31 (PECAM-1)	R&D	Cat#AF3628<br>RRID: AB_2161028
rat monoclonal anti-CD45 (Leukocyte Common Antigen, Ly-5) (clone 30-F11)	BD Biosciences	Cat#550539<br>RRID: AB_2174426
rabbit monoclonal anti-CD56 (NCAM-1) (clone E7X9M)	Cell Signaling Technology	Cat#99746<br>RRID: AB_2868490
mouse monoclonal anti-CD68 (clone PG-M1)	Agilent	Cat#M0876<br>RRID: AB_2074844
mouse monoclonal anti-CD68-PE (clone Y1/82A)	Biolegend	Cat#333807<br>RRID: AB_1089057

mouse monoclonal anti-CD206-APC-Cy7 (clone 15-2)	Biolegend	Cat#321119<br>RRID: AB_2144932
rat monoclonal anti-F4/80 (clone CI:A3-1)	Abcam	Cat#ab6640<br>RRID: AB_1140040
rabbit monoclonal anti-GAPDH (clone 14C10)	Cell Signaling Technology	Cat#2118<br>RRID: AB_561053
rabbit polyclonal anti-Granzyme K	Novus	Cat#NBP2-49387<br>RRID: AB_2939042
rabbit monoclonal anti-Granzyme K	R&D	Cat#MAB10216<br>RRID: AB_2940929
mouse monoclonal anti-HLA-DR-APC (clone L243)	Biolegend	Cat#307609<br>RRID: AB_314687
rabbit polyclonal anti-IL-23a	LSBio (LifeSpan)	Cat#LS-B573<br>RRID: AB_2280244
rabbit monoclonal anti-Ki67 (clone D3B5)	Cell Signaling Technology	Cat#9129<br>RRID: AB_2687446
mouse monoclonal anti-mast cell tryptase (clone AA1)	Bio-Rad	Cat#MCA1438<br>RRID: AB_322318
rabbit polyclonal anti-neutrophil elastase	Abcam	Cat#ab68672<br>RRID: AB_1658868
rabbit monoclonal anti-PAR-1 (clone E9J9L)	Cell Signaling Technology	Cat#79109<br>RRID: AB_3086831
rabbit polyclonal anti-PCNA	LSBio (LifeSpan)	Cat#LS-B2826<br>RRID: AB_3086830
rabbit polyclonal anti-PCNA (clone FL-261)	Santa Cruz	Cat#sc-7907<br>RRID: AB_2160375
mouse monoclonal anti-phospho p38 MAPK (Thr180/Tyr182) (clone 28B10)	Cell Signaling Technology	Cat#9216<br>RRID: AB_331296
rabbit polyclonal anti-p38 MAPK	Cell Signaling Technology	Cat#9212<br>RRID: AB_330713

mouse monoclonal anti-phospho p44/42 MAPK (ERK1/2) (Thr202/Tyr204) (clone E10)	Cell Signaling Technology	Cat#9106<br>RRID: AB_331768
rabbit monoclonal anti-p44/42 MAPK (ERK1/2) (clone 137F5)	Cell Signaling Technology	Cat#4695<br>RRID: AB_390779
rabbit polyclonal anti-phospho p46/54 (SAPK/JNK) (Thr183/Tyr185)	Cell Signaling Technology	Cat#9251<br>RRID: AB_331659
rabbit polyclonal anti-p46/54 (SAPK/JNK)	Cell Signaling Technology	Cat#9252<br>RRID: AB_2250373
rabbit monoclonal anti-phospho-STAT3 (Ser727) (clone D8C2Z)	Cell Signaling Technology	Cat#94994<br>RRID: AB_2800239
rabbit monoclonal anti-phospho-STAT3 (Tyr705) (clone D3A7)	Cell Signaling Technology	Cat#9145<br>RRID: AB_2491009
rabbit monoclonal anti-STAT3 (clone D3Z2G)	Cell Signaling Technology	Cat#12640<br>RRID: AB_2629499
goat anti-rabbit IgG (H+L), biotinylated secondary antibody	Vector Laboratories	Cat#BA-1000<br>RRID: AB_2313606
goat anti-mouse IgG (H+L), biotinylated secondary antibody	Vector Laboratories	Cat#BA-9200<br>RRID: AB_2336171
goat anti-rat IgG (H+L), biotinylated secondary antibody	Vector Laboratories	Cat#BA-9400<br>RRID: AB_2336202
rabbit anti-goat IgG (H+L), biotinylated secondary antibody	Vector Laboratories	Cat#BA-5000<br>RRID: AB_2336126
donkey polyclonal anti-rabbit IgG (H+L)-Alexa Fluor™ 594 ready probes secondary antibody	Thermo Fisher Scientific	Cat#R37119<br>RRID: AB_2556547
goat anti-rabbit IgG (H+L)-HRP Secondary Antibody	Bio-Rad	Cat#1706515<br>RRID: AB_11125142
goat anti-mouse IgG (H+L)-HRP Secondary Antibody	Bio-Rad	Cat#1706516<br>RRID: AB_2921252
**Biological samples**		

healthy adult skin tissue	University of British Columbia/VGH	N/A
psoriasis adult skin tissue	University of British Columbia/VGH	N/A
**Chemicals, peptides, and recombinant proteins**		
30% Acrylamide/Bis solution 37.5:1	Bio-Rad	Cat#1610158
β-Mercaptoethanol	Gibco	Cat#21985-023
Imiquimod Pump 5% w/w Cream	Taro	Cat#02482983
human recombinant granzyme K protein	Bon Opus	N/A (non-commercial)
Cell Dissociation Buffer – Enzyme-Free, PBS-Based	Gibco	Cat#13151-014
D-(+)-Glucose	Sigma-Aldrich	Cat#G7021
Dimethyl sulfoxide (DMSO)	Sigma-Aldrich	Cat#D8418
Dulbecco's Modified Eagle Medium (DMEM), high glucose	Sigma-Aldrich	Cat#D6429
Dulbecco's Phosphate Buffered Saline (PBS)	Sigma-Aldrich	Cat#D8537
EDTA-free complete protease inhibitor cocktail	Roche	Cat#11873580001
Fetal Bovine Serum (FBS), heat inactivated	Gibco	Cat#12484-028
HEPES solution	Sigma-Aldrich	Cat#H0887
HBSS with 10 mM HEPES, without Phenol Red	StemCell	Cat#37150
HiPerFect® Transfection Reagent	Qiagen	Cat#301704
Hoechst 33342	Thermo Fisher Scientific	Cat#H3570
human recombinant IFN-γ protein	R&D	Cat#285-IF
Keratinocyte Basal Medium 2	Promo Cell	Cat#C-20211
Lipopolysaccharides from *E. coli* (O111:B4) (LPS)	Sigma-Aldrich	Cat#L4391
OptiMEM® Reduced Serum Media	Gibco	Cat#31985-062
Penicillin/Streptomycin	Sigma-Aldrich	Cat#P4333
Propidium Iodide Solution	BioLegend	Cat#421301
Phosphatase Inhibitor Cocktail 'PhosSTOP™'	Roche	Cat#04906845001
Phorbol 12-myristate 13-acetate (PMA)	Sigma-Aldrich	Cat#P8139
Protease Inhibitor Cocktail, EDTA-free 'cOmplete™'	Roche	Cat#11873580001

RNase AWAY™ Surface Decontaminant	Thermo Fisher Scientific	Cat#7002
RPMI-1640 Medium	Sigma-Aldrich	Cat#R8758
Sodium Pyruvate	Gibco	Cat#11360-070
Serum, Donkey	Sigma-Aldrich	Cat#D9663
Serum, Goat	Sigma-Aldrich	Cat#G9023
Serum, Horse	Sigma-Aldrich	Cat#H0146
Serum, Rabbit	Sigma-Aldrich	Cat#R9133
TEMED	Bio-Rad	Cat#1610801
Trypan Blue Stain (0.4%)	Gibco	15250-061
U0126 ethanolate	Sigma-Aldrich	Cat#U120
Critical commercial assays		
RNeasy Mini Kit	Qiagen	Cat#74104
First strand cDNA Synthesis Kit	Origene	Cat#NP100042
Pierce™ BCA Protein Assay Kit	Thermo Fisher Scientific	Cat#23225
Mouse IL-12p70 Quantikine ELISA Kit	R&D	Cat#M1270
Mouse IL-17 Quantikine ELISA Kit	R&D	Cat#M1700
Mouse IL-23 Quantikine ELISA Kit	R&D	Cat#M2300
Human IL-23 Quantikine ELISA Kit	R&D	Cat#D2300B
Deposited data		
Human scRNA-seq gene expression profiles (accession numbers: GSE13355, GSE30999)	This paper; Gene Expression Omnibus	https://www.ncbi.nlm.nih.gov/geo/
Human gene(s) co-expression profiles	This paper; SEEK	http://seek.princeton.edu/
Experimental models: Cell lines		
Human: immortalized keratinocytes (HaCaTs)	CLS	Cat#300493;<br>RRID: CVCL_0038
Human: immortalized monocytes (THP-1s)	ATCC	Cat#TIB-202;<br>RRID: CVCL_0006
Experimental models: Organisms/strains		

Mouse: C57BL/6J (listed as 'WT' in text)	Jackson Laboratories	RRID: IMSR_JAX:000664
Mouse: Granzyme K$^{-/-}$ (listed as 'GzmK KO' in text)	In-house (laboratory of David Granville)	N/A
Oligonucleotides		
siRNA targeting sequence: Human F2R #2 (labelled '#1' in text)	Qiagen	GeneGlobeID: SI00031045
siRNA targeting sequence: Human F2R #6 (labelled '#2' in text)	Qiagen	GeneGlobeID: SI02757412
siRNA targeting sequence: unspecific / AllStars negative control	Qiagen	Cat#1027280<br>GeneGlobeID: SI03650318
Primer pair for human *GAPDH*, Forward: 5'-<br>TGCACCACCAACTGCTTAGC<br>-3' Reverse: 5'- GGCATGGACTGTGGTCATGAG -3'	Invitrogen	Cat#10336022
Primer pair for human *IL-23*, Forward: 5'-TGCAAAGGATCCACCAGGGTCTGA-3' Reverse: 5'-TAGGTGCCATCCTTGAGCTGCTGC-3'	Invitrogen	Cat#10336022
Software and algorithms		
Aperio ImageScope	Leica Biosystems	RRID: SCR_020993<br>https://www.leicabiosystems.com/digital-pathology/manage/aperio-imagescope/
Fiji Image J	ImageJ	RRID: SCR_002285<br>https://fiji.sc
FlowJo	BD Biosciences	RRID: SCR_008520<br>https://www.flowjo.com/solutions/flowjo
GraphPad Prism	GraphPad	RRID: SCR_002798<br>https://www.graphpad.com/
QuPath	University of Edinburgh	RRID: SCR_018257<br>https://qupath.github.io/
Other		

Qsonica Ultrasound Sonicator Q125	Qsonica	RRID: SCR_019046
BD LSRFortessa™ Cell Analyzer	BD Biosciences	RRID: SCR_019600
BioRad T100 Thermal Cycler	BioRad	RRID: SCR_021921
LI-COR Odyssey Fc Imaging System	LI-COR	RRID: SCR_023227
